# Paeonol induces cytoprotective autophagy via blocking the Akt/mTOR pathway in ovarian cancer cells

**DOI:** 10.1038/s41419-019-1849-x

**Published:** 2019-08-13

**Authors:** Likun Gao, Zhi Wang, Danhua Lu, Jinling Huang, Jin Liu, Li Hong

**Affiliations:** Department of Obstetrics and Gynaecology, Renmin Hospital of Wuhan University, Wuhan, People’s Republic of China

**Keywords:** Drug development, Autophagy

## Abstract

Paeonol (Pae), a phenolic acid compound isolated from the Moutan Cortex, was previously demonstrated to exert multiple anticancer effects. The rational control of autophagy has been considered a potential treatment strategy for epithelial ovarian cancer. However, whether Pae induces autophagy and the relationship between its antitumour activities and autophagy in epithelial ovarian cancer are still unclear. In this study, we found that Pae induced not only antiproliferation activity and apoptosis but also autophagy, and complete autophagic flux was observed in A2780 and SKOV3 cells. In addition, combination treatment with Pae and an autophagy inhibitor (3-methyladenine and hydroxychloroquine) showed significant synergetic effects on inhibiting cell viability and promoting apoptosis in vitro and in the A2780 xenograft model, without severe side effects, which was often had by cisplatin. These results indicate that autophagy induced by Pae has a cytoprotective role in both A2780 and SKOV3 cells. Mechanistically, we found that Pae inhibited the protein kinase B(Akt)/mammalian target of rapamycin (mTOR) pathway. Furthermore, when combined with the inhibitors MK2206 and rapamycin to inhibit Akt and mTOR kinase activity, Pae-induced autophagy was increased. Taken together, our results demonstrate that Pae induced cytoprotective autophagy by inhibiting the Akt/mTOR pathway in ovarian cancer cells. Thus, the strategy of combining Pae with an autophagy inhibitor to block Akt/mTOR-dependent autophagy could enhance the antitumour activity of Pae and warrants further application for the treatment of ovarian cancer.

## Introduction

Epithelial ovarian cancer (EOC) continues to be the most frequent gynaecologic malignancy, and it ranks as the fifth leading cause of cancer-related mortality among women worldwide^1^. Although treatment for EOC, including surgery and platinum-based chemotherapy, has improved, the overall survival rate of patients remains at ~40%, with a devastating diagnosis^2^, and 80% of these patients who receive standard treatment will relapse and die due to chemoresistance^3^. Acquired chemoresistance remains a major obstacle for the cure of EOC, and novel effective treatments are still urgently needed. In particular, the multidrug combination strategy is considered a promising approach in cancer treatment^4,5^.

Natural active ingredients originating from Chinese herbal medicines have been indicated to be beneficial in the prevention and treatment of cancer for hundreds of years^6^. Paeonol (Pae; 2′-hydroxy-4′-methoxyacetophenone), a phenolic acid compound derived from the root bark of the Moutan Cortex (*Paeonia suffruticosa*)^7^, has been reported to possess all types of potent properties, including anti-inflammatory^8^, antioxidant^9^, immune regulatory activity^10^, and reverse chemoresistance^11^. Recently, Pae was shown to exhibit favourable anticancer activities in ovarian cancer cells^12,13^ and other types of cancer cell lines, such as prostate cancer^14^, melanoma^15^, lung cancer^16^, gastric cancer^17^, and colon cancer^18^. Although the antitumour activity of Pae has been suggested by cumulative evidence, the detailed underlying mechanisms have not been investigated. In particular, the effect of Pae on autophagy activity in tumour cells and the internal connection between autophagy and antineoplastic activity are unclear.

Autophagy, also known as type II programmed cell death (PCD), is a key intracellular degradative process that is generally characterised beginning with autophagosome formation, vesicle fusion, and autolysosome formation, and ultimately participates in recycling to sustain cellular metabolism and cellular homoeostasis^19^. A dysfunction in autophagy closely contributes to the pathogenesis of diverse disease manifestations, such as neurodegenerative diseases, metabolic disorders, microbial infections, and cancers^20,21^. Similar to serving as a two-edged weapon in cancer development, current genetic and pharmacological studies have demonstrated that autophagy exerts a paradoxical role in antineoplastic therapy. In addition to enhancing the anticancer activity of chemotherapy or radiotherapy by inducing autophagic cell death^22,23^, autophagy-dependent antiapoptosis responses induced by chemotherapeutic agents have been shown in a growing number of studies^24,25^, causing adverse effects on antitumour treatment via multiple pathways, including inhibition of the Akt/mTOR signalling pathway^22,24–26^.

The classical Akt/mTOR signalling transduction pathway regulates many cancer development processes, including proliferation, apoptosis, metabolism, chemoresistance, and genomic instability^27^, and it is the most frequently dysregulated cellular pathway in human cancers^28^, including ovarian cancer^29,30^. Furthermore, the Akt/mTOR pathway is recognised as a key regulatory signal for autophagy^31–33^. Existing studies indicate that the Akt/mTOR pathway negatively regulates autophagic processes^33^. In addition, inhibition of the Akt/mTOR pathway in various cells can cause different biological effects that can activate autophagic cell death^22,34^ in many antitumour drugs or induce cytoprotective autophagy^24,35^. However, the role of the Akt/mTOR signalling pathway in Pae-induced autophagy remains unknown, and its effect on autophagy in promoting cell death or cytoprotection needs further investigation.

Here, we demonstrate that Pae shows anticancer activity in vitro and in animal experiments. Furthermore, our research indicated a cytoprotective role for autophagy in A2780 and SKOV3 cells. Finally, the underlying mechanisms of autophagy induced by Pae in A2780 and SKOV3 cells was investigated by considering the Akt/mTOR pathway as a possible target.

## Results

### Pae exhibits an antiproliferative effect in A2780 and SKOV3 cells

**Fig. 1:**
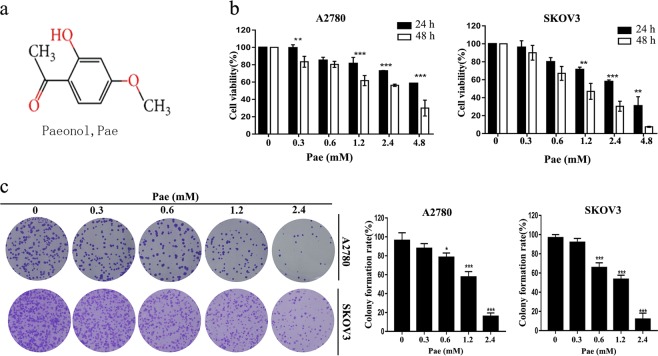
Pae exhibits an antiproliferation effect in A2780 and SKOV3 cells. **a** The molecular structure of Pae. **b** Cells were incubated with various concentrations (up to 4.8 mM) of Pae for 24 and 48 h, and cell viability was then determined by the CCK-8 assay. 0 mM Pae is the DMSO vehicle. **c** Pae inhibited the colony formation ability of A2780 and SKOV3 cells. All data are representative of three independent experiments. Bars, S.E.M.; *P < 0.05, **P < 0.01, ***P < 0.001

As shown in Fig. 1a, the chemical structure of Pae is displayed. First, different concentrations of Pae were incubated with A2780 and SKOV3 cells for the indicated times to assess whether Pae inhibits cell growth. As shown in Fig. 1b, CCK-8 assay data suggest that the proliferation of both A2780 and SKOV3 cells, which respond to Pae treatment, decreases significantly in dose- and time-dependent manners. In addition, whether Pae was toxic to normal ovarian epithelial cell IOSE80 was examined. The results showed that the test doses of Pae were markedly less cytotoxicity toward human normal ovarian epithelial cell IOSE80 (Supplementary Fig. S1). Next, in both A2780 and SKOV3 cells, the inhibitory effects of Pae on cell growth were evaluated by using a colony formation assay. As shown in Fig. 1c, we treated cells with various doses of Pae, and the results showed that colony formation in both cell lines was significantly inhibited by a concentration of 1.2 and 2.4 mM Pae. The IC_50_ of Pae was about 1.2 mM in both A2780 and SKOV3 cells at 48 h, respectively, thus a concentration of 1.2 mM was applied in subsequent experiments. Collectively, the potential antiproliferative effect of Pae was indicated in ovarian cancer cells, but Pae presented less cytotoxic against the normal ovarian epithelial cell.

### Pae-induced ovarian cancer cell apoptosis

**Fig. 2:**
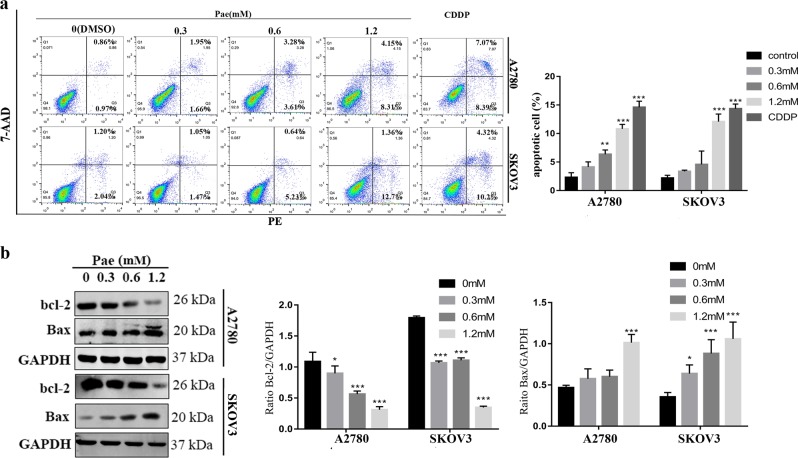
Pae-induced ovarian cancer cell apoptosis. **a** A2780 and SKOV3 cells were treated with Pae at various concentrations or cisplatin (CDDP, 10 μM, as positive control) for 24 h, and then apoptotic cells were detected with the annexin V-PE/7-AAD kit and analysed by flow cytometry. **b** A2780 and SKOV3 cells were treated with Pae at various concentrations for 24 h, and the expression levels of Bcl-2 and Bax were compared by western blot analysis. GAPDH was included as a loading control. All data are representative of three independent experiments. Bars, S.E.M.; *P < 0.05, **P < 0.01, ***P < 0.001

Next, we assessed whether Pae treatment in ovarian cancer cells resulted in cellular apoptosis. Annexin V-PE/7-AAD double staining was performed by flow cytometric analysis. Consistent with our previous data^12^, an increase in both early and late apoptosis in A2780 and SKOV3 cells was induced in a dose-dependent manner after Pae treatment, and the results showed that there were slightly less apoptotic cells observed in Pae-treated ovarian cancer cells when compared to cisplatin-treated (IC_50_ value was about 10 μM) positive control cells (Fig. 2a). Moreover, western blot analysis supported the above data. As shown in Fig. 2b, compared with the control group, the expression of Bcl-2 was significantly decreased; conversely, the expression of Bax protein had obviously increased after Pae treatment. We also examined whether Pae treatment in normal ovarian epithelial cell IOSE80 resulted in cellular apoptosis. The results suggested that the apoptosis rate was not obviously increased after Pae treatment (Supplementary Fig. S2). All these data demonstrated that Pae was effective in inducing cellular apoptosis in ovarian cancer cells but not in normal ovarian epithelial cell.

### Pae induces autophagy in ovarian cancer cells

**Fig. 3:**
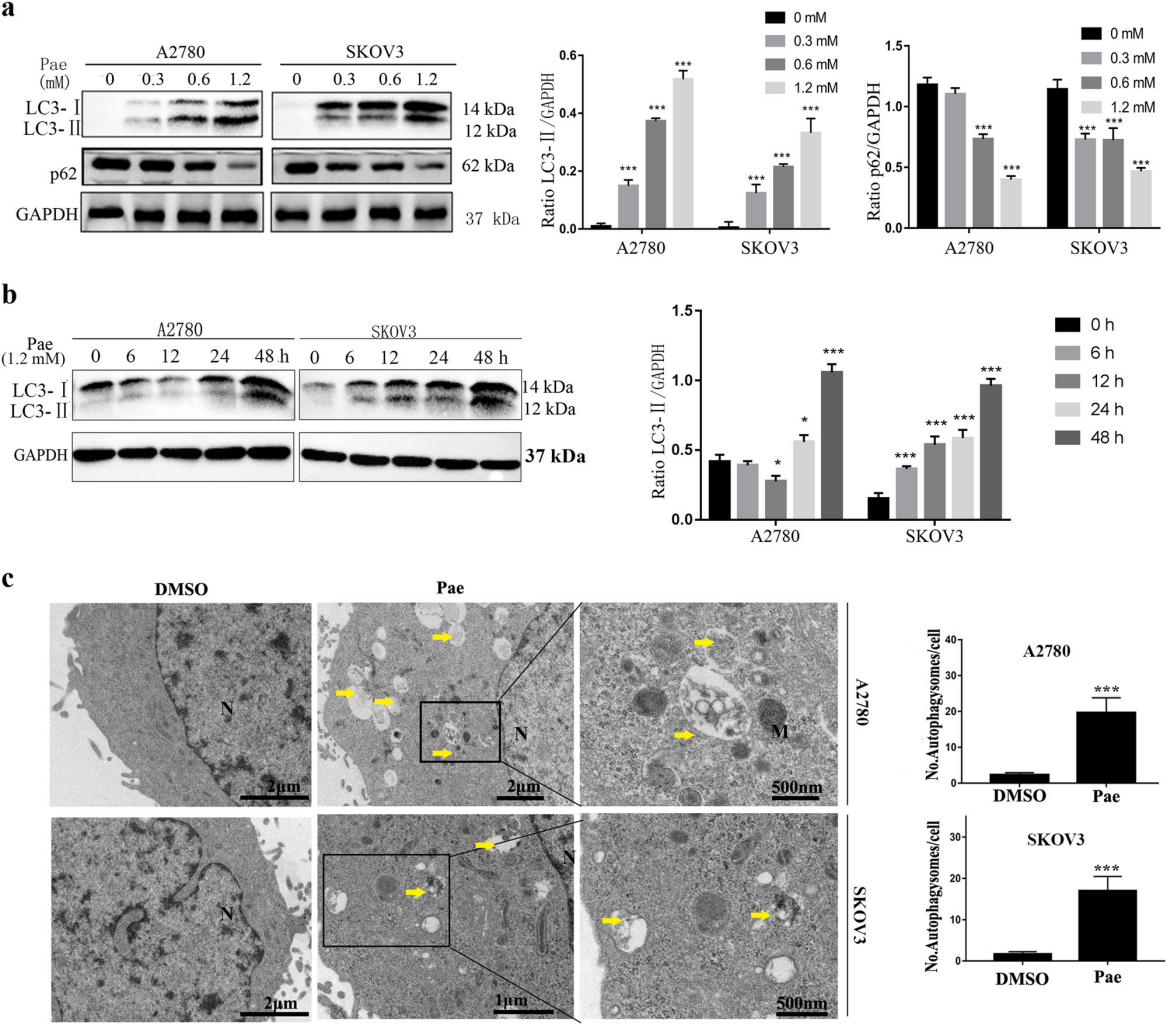
Pae induces autophagy in ovarian cancer cells. A2780 and SKOV3 cells were treated with the indicated concentrations of Pae for 24 h (**a**) or incubated with Pae (1.2 mM) for different times (**b**), and LC3 and p62 were analysed by western blot. GAPDH was included as a loading control. **c** Ultrastructural features of A2780 and SKOV3 cells treated with Pae (1.2 mM) for 24 h were analysed by electron microscopy. Typical images of the nucleus (N), mitochondria (M), and autophagosomes (yellow arrows) are shown at high magnification. The number of autophagosomes in A2780 and SKOV3 cells is presented. Twenty cross-sections were counted in each experiment. All data are representative of three independent experiments. Bars, S.E.M.; *P < 0.05, **P < 0.01, ***P < 0.001

To investigate whether Pae simultaneously induced autophagy and exerted antitumour activity in A2780 and SKOV3 cells, we performed western blot analysis to analyse the classical autophagy markers, the amount of LC3-II conversion and p62. As presented in Fig. 3a and b, LC3-II expression increased in a dose- and time-dependent manner in A2780 and SKOV3 cells after Pae treatment, and the increased level of LC3-II conversion in Pae-treated cells was significantly increased not only following treatment with 0.6 and 1.2 mM Pae but also after treatment for 24 and 48 h. Furthermore, in A2780 and SKOV3 cells, the expression of p62 decreased after Pae treatment in a dose-dependent manner. To examine the formation of autophagosomes after Pae treatment by transmission electron microscopy (TEM), A2780 and SKOV3 cells were treated with Pae (1.2 mM) for 24 h, and large amounts of autophagosomes (indicated by yellow arrows in Fig. 3c) were easily observed compared to the DMSO-treated control cells. Collectively, our data demonstrate that Pae induces autophagy in ovarian cancer cells.

### Pae activates autophagy flux in ovarian cancer cells

**Fig. 4:**
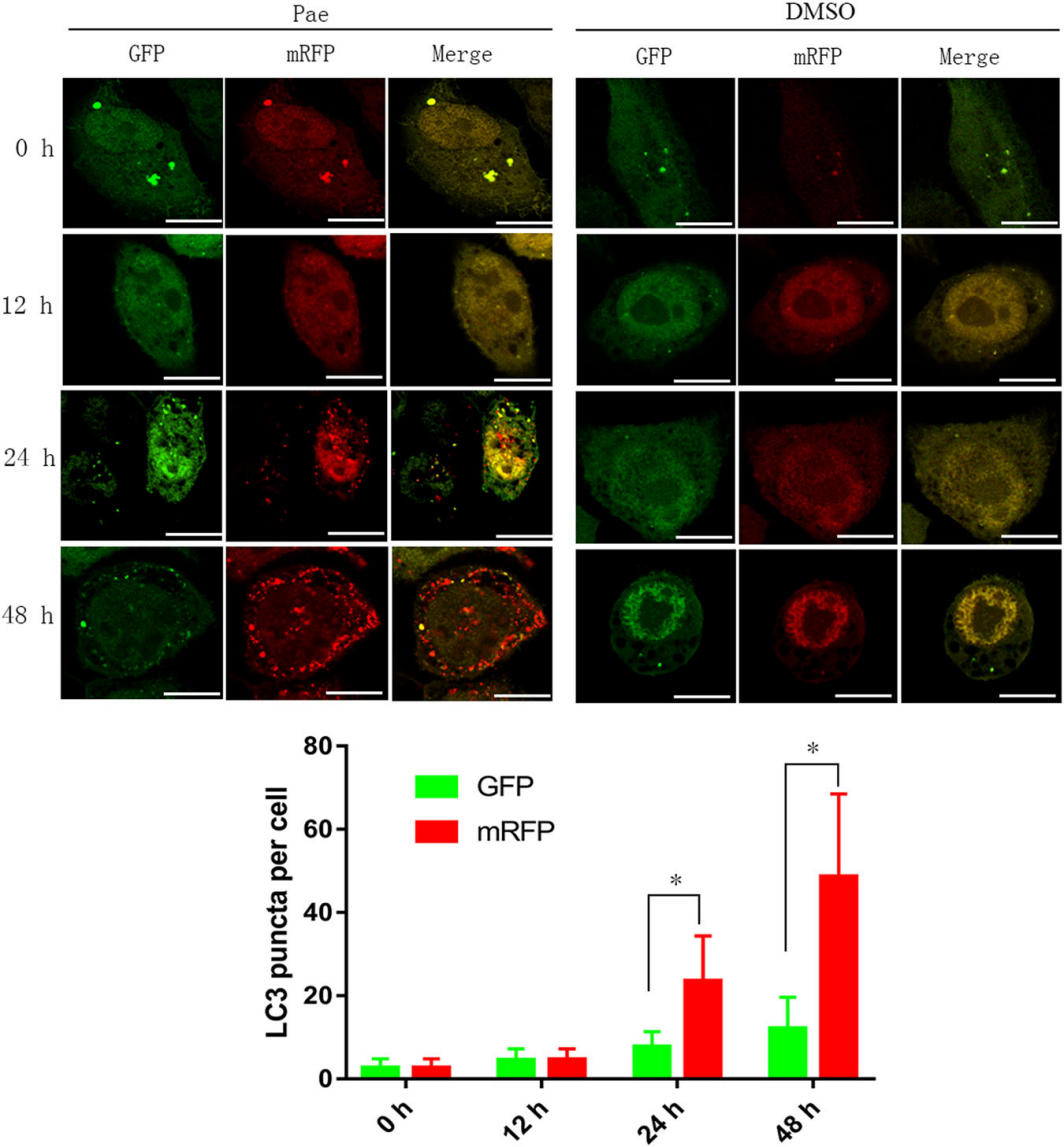
Pae induces the appearance of autophagy flux in ovarian cancer cells. A2780 cells overexpressing mRFP-GFP-LC3 were treated with 1.2 mM Pae (left) or DMSO (right) for the indicated times and then subjected to confocal microscopy. Scale bar: 10 μm. The average numbers of green and red LC3 dots per cell in each condition were quantified, and over 30 cells were counted in each condition. All data are representative of three independent experiments. Bars, S.E.M.; *P < 0.05

To dynamically visualise LC3-labelled cytoplasmic vacuolation to further clarify whether the complete progression of autophagy was affected by Pae, a tandem mRFP-GFP-LC3 adenovirus was transfected into A2780 and SKOV3 cells. Compared with the relatively stable mRFP signal, GFP is more sensitive to the acidic lysosome. Therefore, the tandem mRFP-GFP-LC3 reporter was conveniently used to monitor and quantify autophagic flux inside cells^31,36^. As shown in Fig. 4, SKOV3 and A2780 cells treated with Pae (1.2 mM) for 12 h had slightly accumulated detectable yellow autophagic LC3 puncta (mRFP^+^/GFP^+^) inside the cytoplasm compared with untreated controls, in which only two to five yellow spots were observed. More importantly, red puncta (mRFP^+^GFP^−^) containing mRFP-LC3 became predominantly visible rather than green puncta (mRFP^−^GFP^+^) in cells after 24 and 48 h of Pae stimuli. This result suggests that Pae treatment can accumulate both autophagosomes and autolysosomes, consistent with the western blot results. The tandem fluorescent markers observed and quantified by confocal microscopy strongly confirmed that Pae activated complete autophagic flux.

### Autophagy inhibitors enhance Pae-induced apoptosis and growth inhibition

**Fig. 5:**
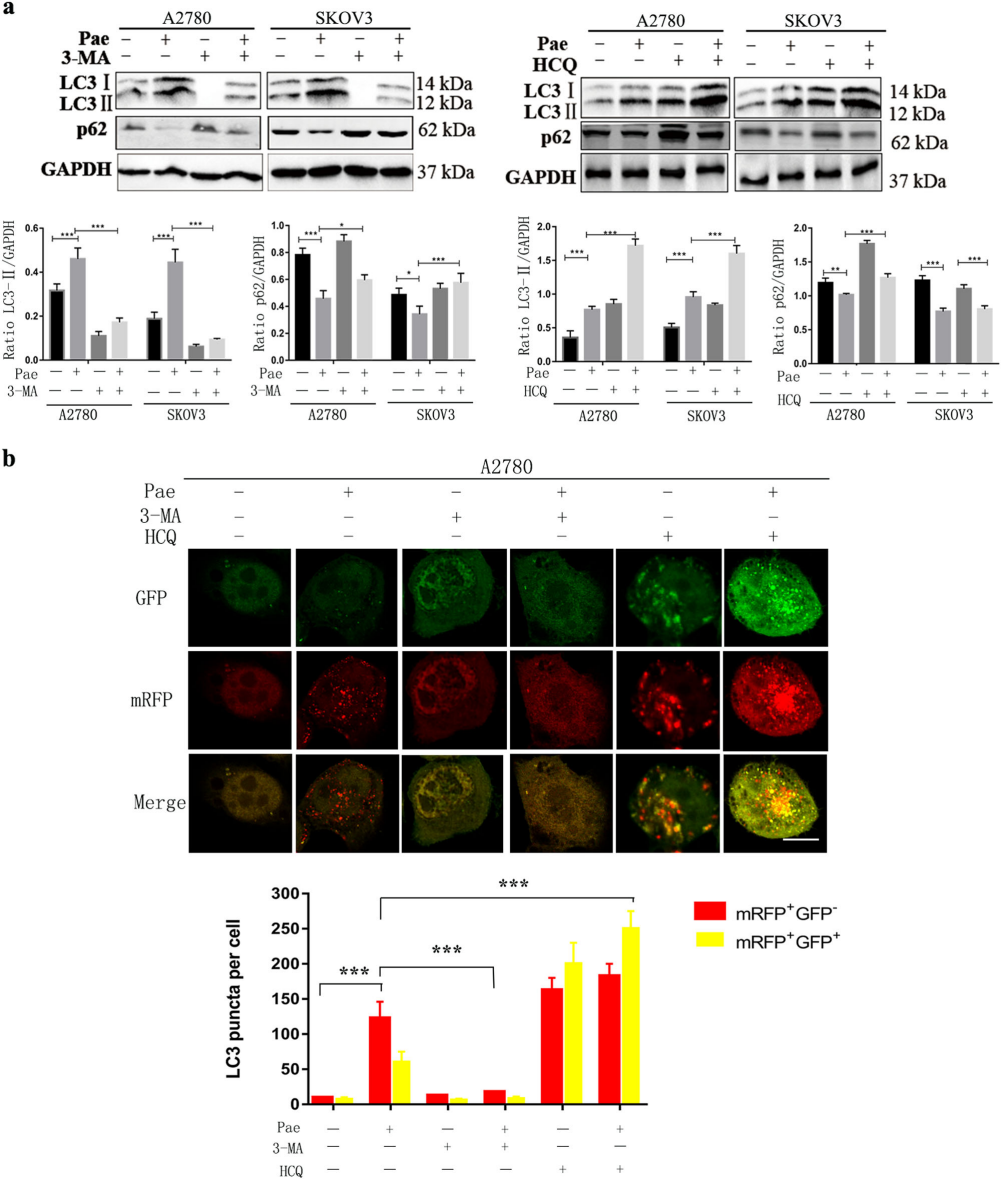
Pae-induced autophagy could be inhibited by autophagy inhibitors. Both A2780 and SKOV3 cells were pre-treated with an inhibitor of autophagy (3-MA or HCQ) for 1 h and then exposed to Pae (1.2 mM) for another 24 h. **a** Western blot analysis of LC3 and p62 expression levels in A2780 and SKOV3 cells. GAPDH was used as a loading control. **b** A2780 cells overexpressing mRFP-GFP-LC3 were treated with 1.2 mM Pae in combination with 3-MA or HCQ, and the cells with mRFP-GFP-LC3 punctate dots (yellow, green, and red dots) were examined. Positive signals were defined if the cell had five or more LC3 puncta in the cytoplasm. The numbers of red and yellow LC3 dots per cell were counted under a fluorescence microscope. Scale bar: 10 μm. All data are representative of three independent experiments. Bars, S.E.M.; *P < 0.05, **P < 0.01, ***P < 0.001

**Fig. 6:**
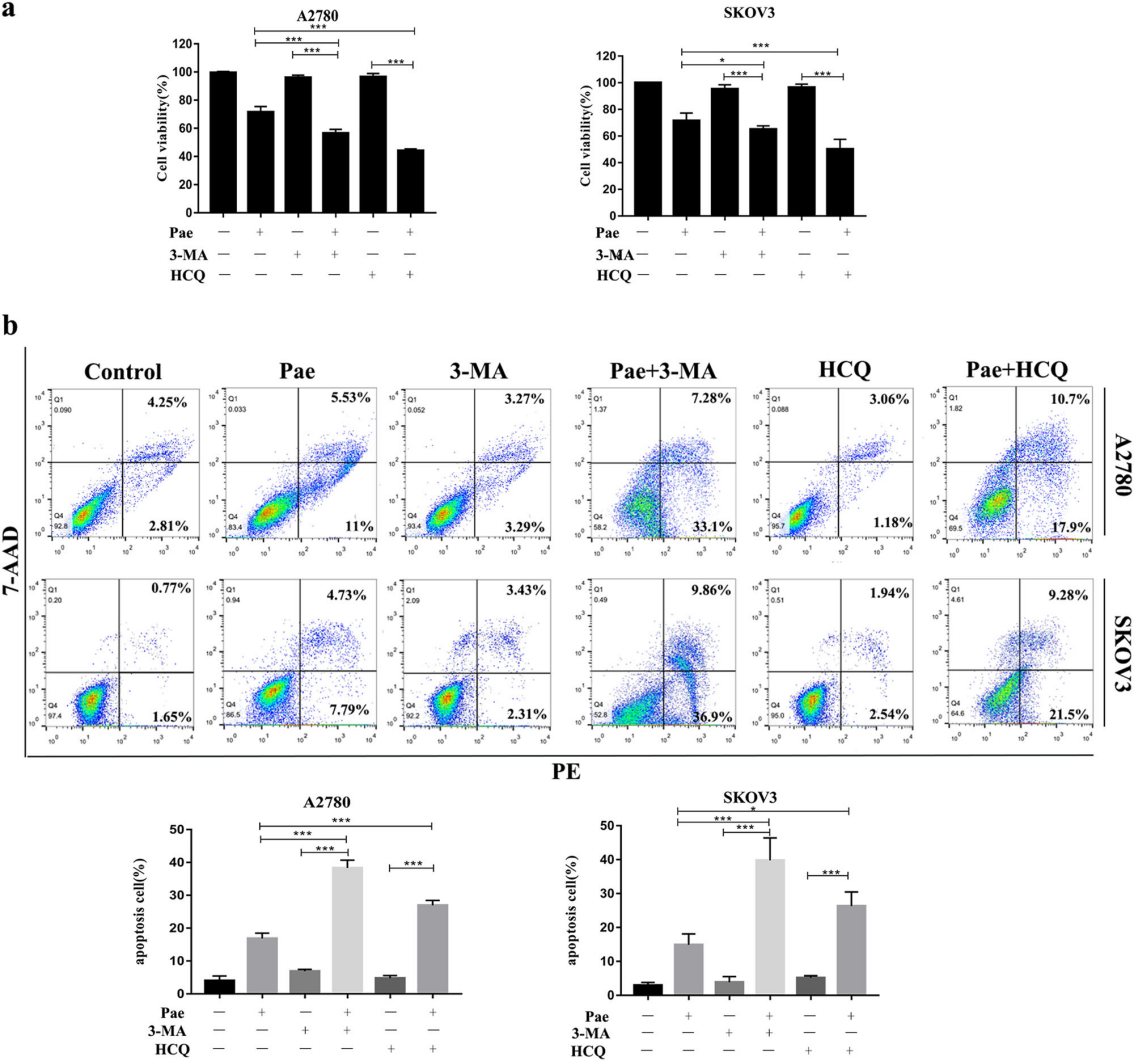
Autophagy inhibitors enhance Pae-induced apoptosis and growth inhibition. **a** Cell viability was determined by the CCK-8 assay. **b** A2780 and SKOV3 cells were treated with Pae alone or together with 3-MA or HCQ for 24 h, and then apoptotic cells were detected with the annexin V-PE/7-AAD kit and analysed by flow cytometry. All data are representative of three independent experiments. Bars, S.E.M.; *P < 0.05, **P < 0.01, ***P < 0.001

Accumulating evidence suggests that autophagy may result in different forms of effects, such as cytoprotective, cytostatic, cytotoxic, and nonprotective, in response to chemotherapy or radiation^37^. To evaluate whether Pae-induced autophagy is related to cell viability and apoptosis, we used two autophagy inhibitors, 3-methyladenine (3-MA) and hydroxychloroquine (HCQ), to block the autophagy process. First, western blot data showed that 3-MA attenuated autophagy induced by Pae, decreased LC3-II protein expression and increased p62 protein levels (Fig. 5a). HCQ induced LC3-II and p62 protein accumulation, consistent with previous studies^38^ (Fig. 5a). Furthermore, we used confocal microscopy to analyse mRFP and GFP LC3 puncta. As shown in Fig. 5b, 3-MA could significantly inhibit autolysosome accumulation in A2780 cells after exposure to Pae, and HCQ could accumulate yellow autophagic LC3 puncta (mRFP+/GFP+). In addition, we observed whether the antitumour effects in ovarian cancer cells induced by Pae could be enhanced by autophagy inhibitors. Compared with Pae treatment alone, CCK-8 assays showed that the combination of 3-MA and HCQ with Pae could strengthen the inhibitory effect of Pae on cell viability (Fig. 6a). Moreover, annexin V-PE/7-AAD assays showed that the combination of Pae with 3-MA or HCQ also significantly increased the number of apoptotic ovarian cancer cells (Fig. 6b). Therefore, the combination treatment of Pae with 3-MA and HCQ enhanced the antitumour effects in ovarian cancer cells.

### Inhibition of the Akt/mTOR signalling pathway is required for Pae-induced cell autophagy

**Fig. 7:**
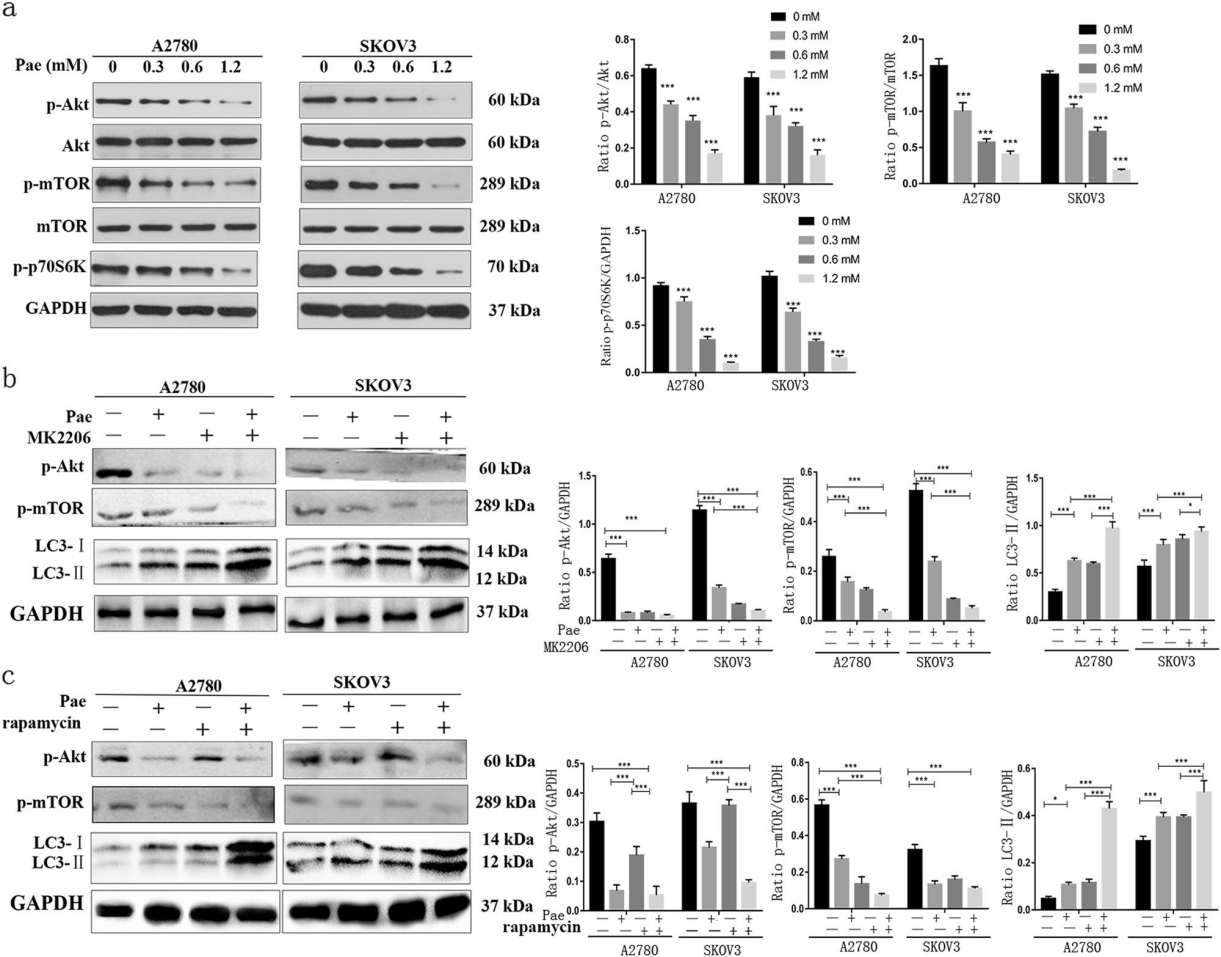
Pae induces autophagy through inhibition of the Akt/mTOR pathway. **a** A2780 and SKOV3 cells were treated with different concentrations of Pae for 24 h. Cell lysates were used to detect the following signal-related proteins: Akt (S473), mTOR (S2448) and their phosphorylated counterparts, p-P70S6K (S424/T421), by western blot analysis. **b** Cells were treated with or without Pae (1.2 mM) in combination with MK2206 for 24 h. Phosphorylated Akt (p-Akt), p-mTOR, LC3-I/II, and LC3 were detected by western blot analysis. **c** Cells were treated with or without Pae (1.2 mM) in combination with rapamycin for 24 h. p-Akt, p-mTOR, and LC3-I/II were detected by western blot analysis. GAPDH was used as a loading control. All data are representative of three independent experiments. Bars, S.E.M.; *P < 0.05, **P < 0.01, ***P < 0.001

Cumulative evidence suggests that Pae regulates the Akt signalling pathway in ovarian cancer cells^12,39^, and in response to external stimuli, the Akt/mTOR pathway has a critical role in autophagy and apoptosis^32,35^. To ascertain whether the Akt/mTOR pathway has an important role in Pae-induced autophagy, we investigated key proteins related to the Akt/mTOR pathway in Pae-treated ovarian cancer cells by western blot analysis. First, the phosphorylated levels of Akt, mTOR, and p70S6K were analysed. As noted in Fig. 7a, compared with the untreated control, treatment with Pae did not significantly change total Akt or mTOR expression, whereas it significantly decreased p-Akt, p-mTOR, and p-p70S6K protein expression in A2780 and SKOV3 cells in a dose-dependent manner. Furthermore, to further investigate the inhibition of the Akt/mTOR signalling pathway in Pae-induced autophagy, we rescued Pae-induced Akt/mTOR inhibition by decreasing p-Akt with MK2206 and analysed LC3-II conversion in Pae-treated ovarian cancer cells. Our finding shows that LC3-II conversion was significantly increased in Pae-treated cells (Fig. 7b). Moreover, rapamycin (an mTOR inhibitor) significantly reduced p-mTOR expression and increased LC3-II conversion in Pae-treated cells (Fig. 7c). Collectively, these findings illustrate that inhibition of the Akt/mTOR pathway is required for Pae-induced autophagy in ovarian cancer cells.

### Combination treatment with Pae and hydroxychloroquine enhances antitumour activities in a xenograft animal model

**Fig. 8:**
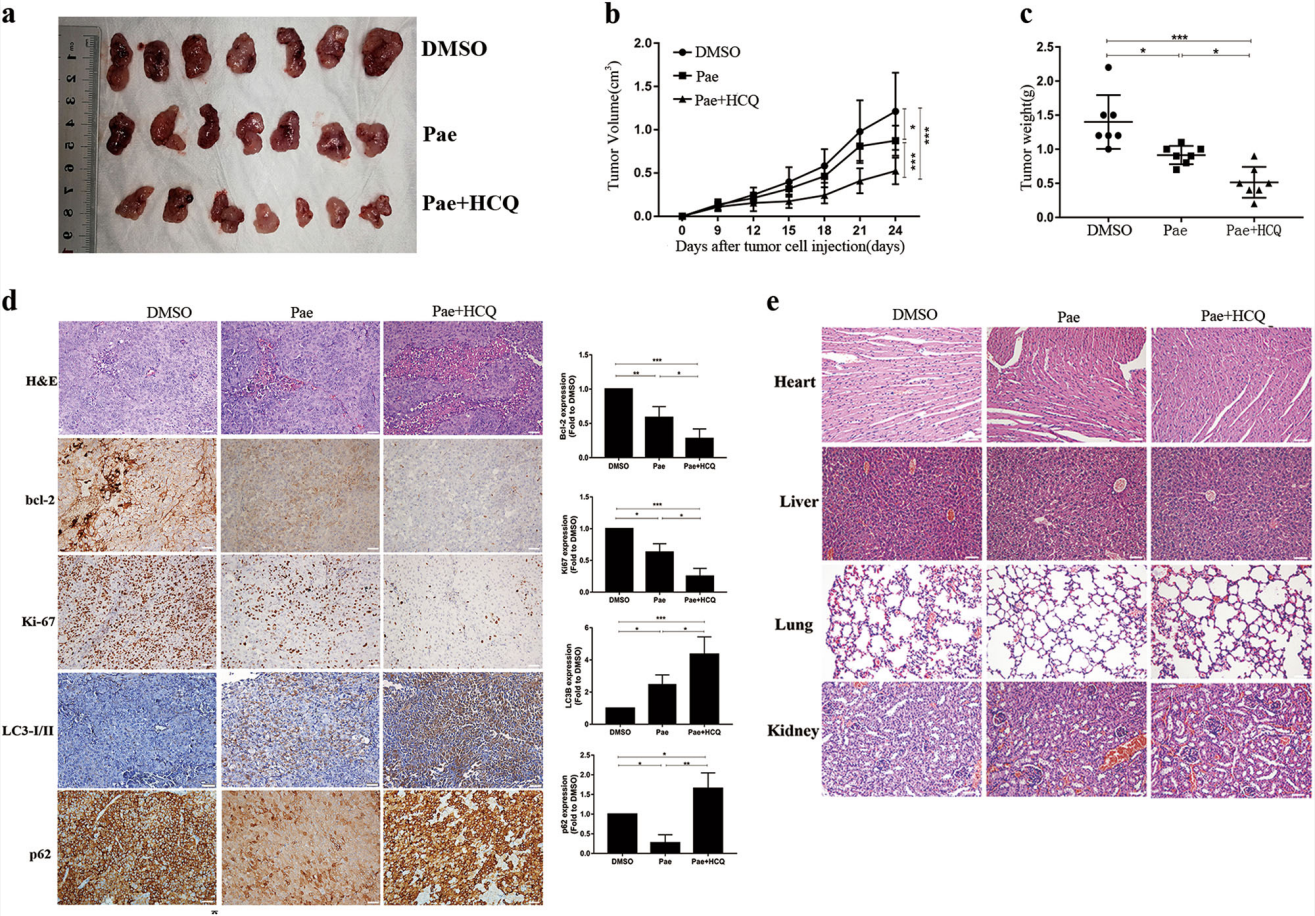
Combination therapy with Pae and hydroxychloroquine enhances antitumour activities in a xenograft animal model. **a** Representative images of subcutaneous tumours after treatment (*n* = 7). **b** The volume and **c** weight of tumours in the Pae and Pae + HCQ groups were significantly less than those in the DMSO group. Tumour volumes at different time points. **d** Bcl-2, Ki-67, LC3-I/II, and p62 in Pae and Pae + HCQ groups tumour tissues compared with the control group were detected by IHC staining. H&E staining (upper panel) and IHC analysis of bcl-2, Ki-67, LC3-I/II, and p62 expression in vivo (middle and lower panels). Scale bar: 50 μm. Original magnification: ×20. **e** H&E staining of important organs. Scale bar: 50 μm. Original magnification: ×20. Data are presented as the mean ± SD (*n* = 7). Bars, S.E.M.; *P < 0.05; **P < 0.01; ***P < 0.001

Finally, we generated a xenograft tumour model to determine whether Pae induced anticancer activity in vivo. The xenograft mice were treated with DMSO control (without Pae), Pae (40 mg/kg)^10,16^, or a combination of Pae (40 mg/kg) and HCQ (60 mg/kg)^40^. As shown in Fig. 8a–c, tumour volume and weight were reduced in the Pae and Pae + HCQ groups, especially in the Pae + HCQ group, and there were significant differences between each group. Our data confirmed the in vitro results and strongly indicate that combination treatment with the autophagy inhibitor HCQ enhances the antitumour activities of Pae. Compared with the untreated control, H&E sections of tumour tissue showed large areas of necrosis. Immunohistochemistry (IHC) staining of tumour tissue revealed decrease in levels of Ki-67 and Bcl-2 protein expression as well as increase in level of LC3-I/II protein in Pae-treated and Pae + HCQ-treated tumour tissues, whereas the protein of p62 expression was decreased in Pae-treated tumour tissues but increased in Pae + HCQ-treated tumour tissues compared with the control group (Fig. 8d). In addition, the protein expression of bcl-2, Ki-67, LC3-I/II and p62 between Pae and Pae + HCQ groups was significant differences. Furthermore, body weight of mice in the Pae group and Pae + HCQ group maintained normal gain during the treatment (Supplementary Fig. S3). And H&E staining of organs revealed no significant major organ-related toxicity in the combined groups compared with the untreated control (Fig. 8e). These data suggest that combination therapy of Pae and the autophagy inhibitor HCQ enhances antitumour activity in vivo and has low levels of organ-related toxicity, and further confirmed the in vitro results related autophagy.

## Discussion

**Fig. 9:**
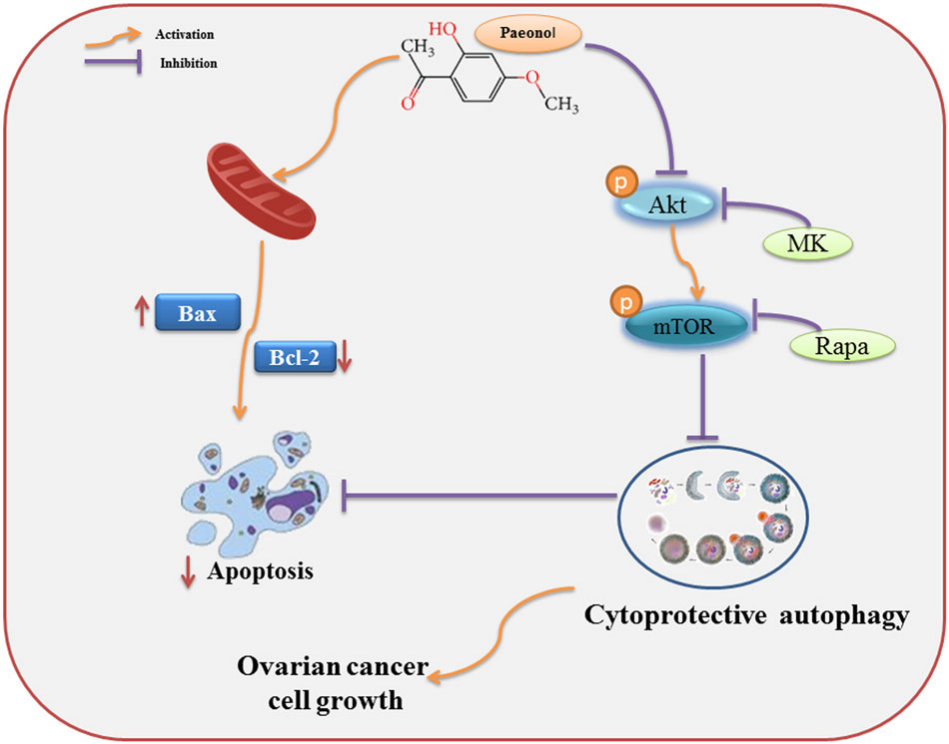
Schematic representation of the mechanism of Pae induces cytoprotective autophagy in ovarian cancer cells. Paeonol induces cytoprotective autophagy via blocking the Akt/mTOR pathway in ovarian cancer cells. MK MK2206; Rapa rapamycin

Growing evidence has proven that many anticancer drugs can induce autophagy in cancer cells, and the effects induced by autophagy in response to stress induced by chemotherapy or radiation can be divided into playing a prodeath or prosurvival role, which contributes to the anticancer efficacy of these drugs and drug resistance^37^. In cancer cells, autophagy contributes to chemotherapy resistance through its cytoprotective effect^41^. Hence, interfering with cytoprotective autophagy helps to strengthen drug susceptibility. Therefore, the rational control of autophagy is of great significance for cancer treatment. Pae has been isolated from the Moutan Cortex, and cumulative evidence suggests that it has effective antitumour activity against various cancer cells^12,15,16^. However, the properties of Pae regarding antiproliferation and the induction of apoptosis and autophagy and the intrinsic relationships in ovarian cancer cells are unknown (Fig. 9e).

In our study, the data showed that Pae exhibits an antiproliferation effect and apoptosis induction, which is in accord with our previous studies^12^. In addition, owing to cisplatin is the first-line standardised chemotherapy drug for ovarian cancer, it was selected as positive control to verify the exact antitumour activity of Pae. The results that pro-apoptotic activity of Pae was only slightly lower than cisplatin confirmed the effective antineoplastic effect of Pae in ovarian cancer. Further experiment found that Pae could induce autophagy and was observed as a complete autophagic flux in A2780 and SKOV3 cells. Moreover, a disruption in Pae-induced autophagy by 3-MA and HCQ resulted in significant synergistic antitumour effects in ovarian cancer cells, as evidenced by a strengthened inhibitory rate of cell viability and a significant increase in the number of apoptotic ovarian cancer cells. These data strongly suggest that autophagy induced by Pae may have a cytoprotective role in ovarian cancer cells and that the combination treatment of Pae and an autophagy inhibitor may be significant for ovarian cancer therapy if Pae is used in the clinic in the future.

Growing evidence indicates that autophagy and apoptosis have complicated intricate relationships, as evidenced by the fact that they share common regulatory element, including the Akt/mTOR signalling pathway. Autophagy activity can be negatively regulated by mediating the phosphorylation of mTOR^32,42^, and inhibition of the Akt/mTOR signalling pathway by different drugs or conditions can play the role resemble as a double-edged sword: it can inhibit apoptosis^24,25^, or promote apoptosis^26^. Cumulative results also indicated that the Akt-related signalling pathway plays an important role in the antitumour activities of Pae in human cancer. For example, the inhibition of Akt activation exerts antiangiogenic and antimetastatic activities^43^, and downregulation of the PI3K/Akt pathway increases radiation-induced apoptosis^44^. Therefore, the Akt/mTOR signalling pathway may be partly involved in autophagy activated by Pae in ovarian cancer cells. Our current data showed that Pae triggered the inhibition of Akt/mTOR, consistent with our hypothesis. Moreover, MK2206 (an Akt inhibitor) increased LC3-II conversion levels in Pae-treated A2780 and SKOV3 cells. In addition, the combination of rapamycin (an mTOR inhibitor) and Pae in ovarian cancer cells also showed similar results. Collectively, these findings strongly support the hypothesis that suppression of the Akt/mTOR signalling pathway is involved in Pae-triggered protective autophagy. However, autophagy induced by Pae therapy in cancers has regrettably not been reported; therefore, the understanding of the target proteins of Pae-induced protective autophagy is limited and requires further investigation.

Furthermore, to support and verify our experimental results in vitro with more reliable evidence, we tested the effect of Pae combined with HCQ in a xenograft nude mouse model. HCQ rather than the autophagy inhibitors 3-MA and CQ is used in xenograft models because it is less toxic^38^. We found that HCQ significantly increased the inhibition effects of tumour growth by Pae, and neither showed any abnormality in behaviour nor significant major organ-related toxicity which is often had by cisplatin, such as nephrotoxicity, hepatotoxicity, cardiotoxicity, and so on^45^. Thus, interfering with Pae-induced cytoprotective autophagy by HCQ strengthens the antitumour activities of Pae without severe side effects in animal models. Moreover, together our data suggested that Pae may be a relatively effective and safe agent with less side effects in ovarian cancer. Regretfully, there are certain limitations in animal experiment owing to a single dose, and further studies in ovarian cancer animal models as well as in human clinical trials are necessary.

In summary, our study demonstrated that the potential anticancer agent Pae induces cytoprotective autophagy via inhibition of the Akt/mTOR pathway in ovarian cancer cells. Combination therapy with Pae and an autophagy inhibitor enhances the antitumour activities of Pae. These findings unveil the potential anticancer molecular mechanism of Pae and strongly indicate that combination treatment with Pae and an autophagy inhibitor is a new strategy for the treatment of ovarian cancer.

## Materials and methods

### Chemicals and reagents

Paeonol (purity of 99%) was purchased from Sigma-Aldrich Co. (H35803). Pae was dissolved in dimethyl sulfoxide (DMSO, Sigma, D2650) and stored at −20 °C. Other reagent sources are listed below: foetal bovine serum (FBS) and trypsin/EDTA solution (Gibco, Thermo Fisher Scientific, Waltham, MA), Dulbecco’s modified Eagle medium (DMEM), Cell Counting Kit (CCK)-8 (Multisciences Biotech, China), 3-MA and hydroxychloroquine(Sigma-Aldrich, St Louis, MO, USA), MK2206 (MCE, HY-10358), Rapamycin (MCE, HY-10219), and bicinchoninic acid (BCA) protein assay kit (Beyotime Institute of Biotechnology, China).

### Cell culture

The ovarian cancer cell lines A2780 and SKOV3, and human normal ovarian epithelial cell IOSE80 were obtained from the China Center for Type Culture Collection (CCTCC, Wuhan, China). These cell lines were cultured in DMEM supplemented with 10% foetal bovine serum and 1% antibiotics (penicillin and streptomycin) in an incubator and 5% CO_2_ at 37 °C. Logarithmically growing cells were used in all subsequent experiments.

### Colony formation assay

To analyse colony formation, A2780 and SKOV3 cells were seeded at ~500 cells per well in six-well plates, and the medium was changed every 3 days. After 24 h, the cells were exposed to different concentrations (0, 0.3, 0.6, 1.2, 2.4, and 4.8 mM) of Pae and incubated in a humidified atmosphere of 5% CO_2_ at 37 °C for 14 days. The control (0 mM) was incubated with an equal volume of the drug’s vehicle DMSO (the final concentration of DMSO in the medium is <1‰), but the applied concentration did not exhibit a modulating effect on cell growth. Finally, the plates were washed with PBS twice, fixed with methyl alcohol for 15 min, and stained with 1% crystal violet for 5 min. ImageJ software was used to quantify the number of colonies in three independent experiments.

### Cell viability assay

To determine cell viability, cells were plated into 96-well plates at a density of 5000 cells/well. Then, different concentrations of Pae (0, 0.3, 0.6, 1.2, 2.4, and 4.8 mM)were incubated with the cells for 24 and 48 h. Subsequently, the CCK-8 assay was performed by adding 10 µL of CCK-8 reagent to each well, and the plates were incubated for 2 h in an atmosphere of 5% CO_2_ and 37 °C. The plates were measured at 450 nm on a PerkinElmer Victor3 1420 Multilabel Counter (Waltham, MA).

### Flow cytometric analysis of apoptosis

A2780 and SKOV3 cells were seeded in 24-well plates (6 × 10^4^ per well) were treated with Pae (0, 0.3, 0.6, 1.2 mM) and the autophagy inhibitor 3-MA or HCQ for 48 h and then collected and detected using an Annexin V-PE/7-AAD Apoptosis Detection Kit (BD Biosciences, San Diego, CA, USA) for apoptosis analysis. A flow cytometry FACSCalibur system (BD, Franklin Lakes, NJ, USA) was used to analyse cells, and FlowJo software (BD Biosciences) was used for data analysis.

### Transmission electron microscopy

After treatment with Pae (1.2 mM) for 24 h, A2780 and SKOV3 cells were fixed in 4% glutaraldehyde overnight and then fixed with 1% osmium tetroxide. After dehydration in a series of ethanol and infiltration with propylene oxide, samples were embedded. Sections of ~50 nm were cut and double stained with 3% uranyl acetate and lead citrate, and then samples were observed by transmission electron microscopy (HITACHI HT7700, Tokyo, Japan).

### Autophagy flux analysis

For the detection of autophagosomes and autolysosomes, A2780 and SKOV3 cells were transfected with mRFP-GFP-LC3 adenoviral vectors, which were purchased from HanBio Technology (Shanghai, China). Then, the cells were incubated in medium containing the indicated concentrations of Pae (1.2 mM) or/and the autophagy inhibitor for the for indicated times at 37 °C. Autophagic flux observation and mounting were performed with a Zeiss LSM710 confocal microscope (Carl Zeiss).

### Western blot analysis

The preparation of total protein lysates and western blot analysis were performed as described previously^46^. The primary antibody information was as follows: anti-p62 (1:1000; MBL, M162-3), anti-LC3-I/II (1:3000; MBL, PM036), anti-GAPDH (1:10,000; Abcam, ab37168), anti-Bcl-2 (1:200; Abcam, ab32124), anti-Bax (1:1000; Abcam, ab32503), anti-Akt (1:500; Abcam, ab8805), anti-phospho-Akt (1:1000; Proteintech, 10176-2-AP), anti-mTOR (1:10,000; Abcam, ab134903), anti-p-mTOR (1:1000; Abcam, ab137133), and anti-p-p70S6K (1:5000; CST, # 9204 S). Signals that were detected by an Odyssey infra-red imaging system (LI-COR Biosciences, Lincoln) were then quantified by ImageJ software.

### Tumour xenograft study

The ethical committee of the Institutional Animal Care and Use Committee of Renmin Hospital of Wuhan University approved all experimental procedures in this study (ethic number 20190503), and all experimental procedures were handled according to the National Institutes of Health Guidelines for the Care and Use of Animals. Xenograft tumour models were established in female BALB/c nude mice (nu/nu, aged 6–8 weeks) that were purchased from the Beijing Vital River Laboratory Animal Technology Cooperation (Beijing, China). Logarithmic growth phase A2780 cells (1 × 10^7^/0.2 mL) were subcutaneously injected into the left flank of each mouse (seven mice in each group). When the tumour size reached approximately 50 mm^3^ (at about day 9), the mice were treated with DMSO (Pae 0 mg/kg), Pae (40 mg/kg), and Pae (40 mg/kg) + HCQ (60 mg/kg) by intraperitoneal injection once every 2 days and continued for six total times (12 days) and observed to 24 days. Tumour volume was monitored and calculated every 3 days by the following formula: tumour volume = (length × width^2^)/2. Mice were anaesthetised and sacrificed until the experiment was terminated, and tumour tissues were removed for further analysis.

### Histopathology and Immunohistochemistry

Tissue samples isolated from xenograft tumours were subjected to histological analysis. Briefly, tissue samples were first fixed with formalin and then embedded in paraffin, cut into 5 µm sections and stained with H&E for IHC staining. IHC staining was conducted in a DAKO Autostainer system (Dako, Glostrup, Denmark). The primary antibodies used were Bcl-2 (Abcam, ab32124), Ki-67 (Abcam, Cambridge, MA, UK), LC3-I/II (Proteintech, 14600-1-AP), p62 (MBL, M162-3). Primary tumours and major organ sections were stained with H&E. Images were visualised using an Olympus microscope (Japan), and image analysis was performed by Image-Pro Plus 6.0.

### Statistical analysis

Statistical analysis was performed with GraphPad Prism software version 7 (San Diego, CA, USA). One-way ANOVA or one-way ANOVA combined with two-sample equal variance Student’s *t*-test method was performed to determine statistical significance. *P* < 0.05 was considered statistically significant. All data are expressed as the mean ± standard error.

## Supplementary information

Supplyment Figure S1

Supplyment Figure S2

Supplyment Figure S3

Supplementary material
