# Supplementary figures and images for: Paeonol induces cytoprotective autophagy via blocking the Akt/mTOR pathway in ovarian cancer cells
Source: Cell Death Dis. 2019 Aug 13;10(8):609. doi: 10.1038/s41419-019-1849-x (PMC6690917; doi:10.1038/s41419-019-1849-x)

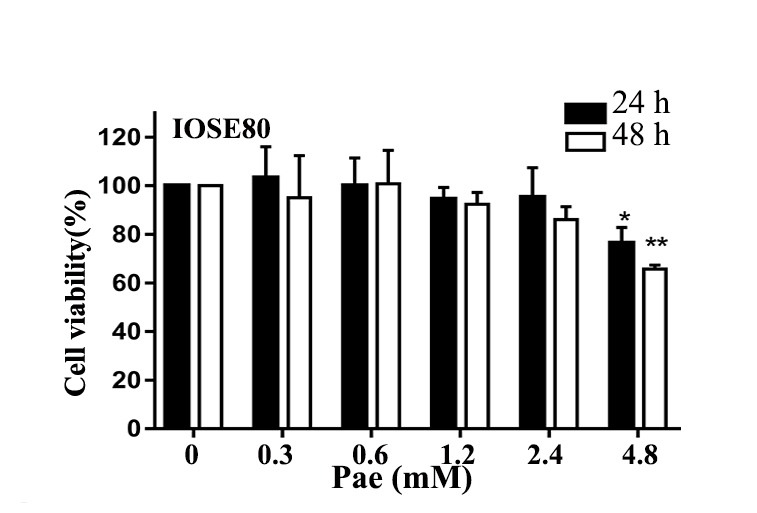

Supplement: Supplementary file 1 — Supplyment Figure S1 [file 41419_2019_1849_MOESM1_ESM.tif]

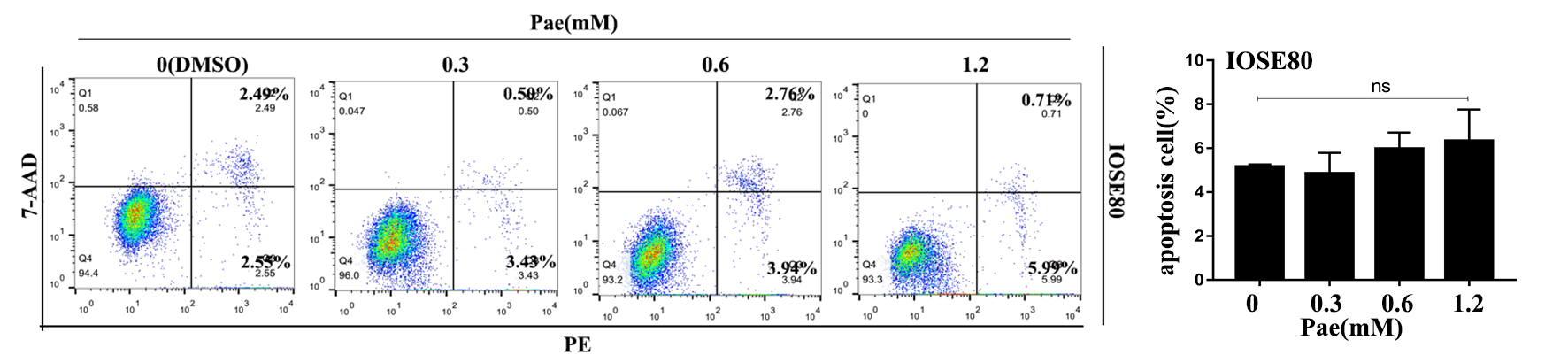

Supplement: Supplementary file 2 — Supplyment Figure S2 [file 41419_2019_1849_MOESM2_ESM.tif]

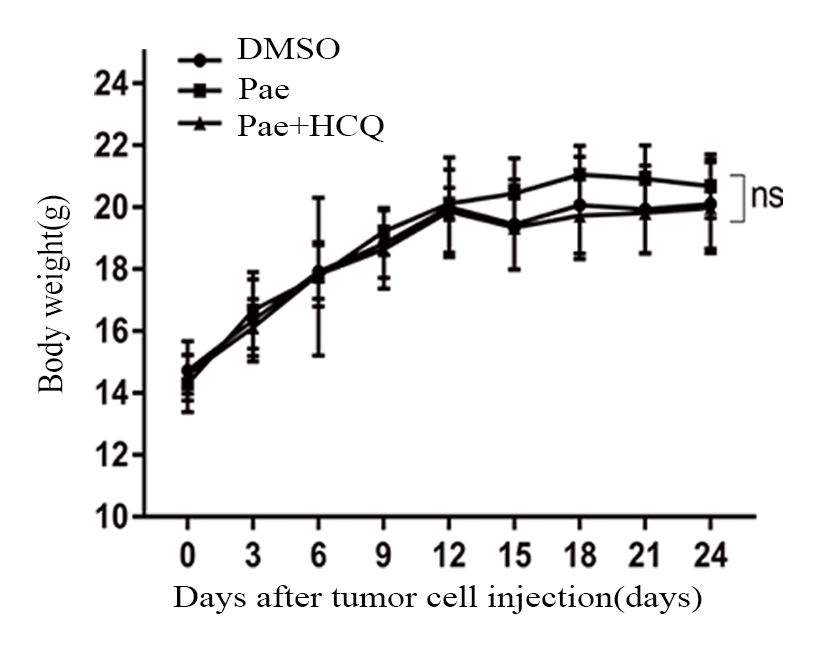

Supplement: Supplementary file 3 — Supplyment Figure S3 [file 41419_2019_1849_MOESM3_ESM.tif]
