# Supplementary material for: Paeonol induces cytoprotective autophagy via blocking the Akt/mTOR pathway in ovarian cancer cells
Source: Cell Death Dis. 2019 Aug 13;10(8):609. doi: 10.1038/s41419-019-1849-x (PMC6690917; doi:10.1038/s41419-019-1849-x)
Supplement: Supplementary file 4 — Supplementary material [file 41419_2019_1849_MOESM4_ESM.docx]

**Supplementary material:**

**Supplementary Figure Legends**

**Fig. S1** Pae presented less cytotoxic against the normal ovarian epithelial cells

Normal ovarian cancer cell IOSE80 were treated with indicated concentrations of Pae for 24 h and 48h. Cell viability was examined by CCK8 assay. The number from the DMSO (0 mM) group was counted as 100%, and those of other groups were relative to that. All data are representative of three independent experiments. Bars, S.E.M.; *P<0.05, **P<0.01, ***P<0.001.

**Fig. S2** Pae did not induce normal ovarian epithelial cell apoptosis

Normal ovarian cancer cell IOSE80 were treated with indicated concentrations of Pae for 24h. Annexin V-PE/7-AAD double staining was performed by flow cytometric analysis for cellular apoptosis.The DMSO (0 mM) group was considered as control, and those of other groups were relative to that. All data are representative of three independent experiments. Bars, S.E.M.; *P<0.05, **P<0.01, ***P<0.001.

**Fig. S3** Combination therapy with Pae and hydroxychloroquine maintains normal weight gain in a xenograft animal model

The body weight of Mice in the Pae group and Pae+HCQ group maintained normal gain throughout treatment**.** The body weight volumes at different time points. Data are presented as the mean ± SD (n＝7). Bars, S.E.M.; **ns** no significance.
